# Multiple Symmetric Lipomatosis: A Diagnostic Dilemma

**DOI:** 10.1155/2013/836903

**Published:** 2013-08-01

**Authors:** Mladen Mimica, Danijel Pravdic, Emina Nakas-Icindic, Maja Karin, Emil Babic, Monika Tomic, Milenko Bevanda

**Affiliations:** ^1^University of Mostar School of Medicine and University of Mostar Clinical Hospital, Bijeli Brijeg bb, 88000 Mostar, Bosnia and Herzegovina; ^2^Department of Physiology, Faculty of Medicine, University of Sarajevo, Čekaluša 90, 71000 Sarajevo, Bosnia and Herzegovina

## Abstract

*Introduction*. Multiple symmetric lipomatosis, or Madelung's disease, is a rare condition which is characterized with large symmetrical accumulation of noncapsulated fat tissue in upper arms, neck, and shoulder areas. The disease etiology is unknown, with the highest incidence in the Mediterranean region. *Case Presentation*. Here, we present the case of Madelung's disease with symmetric fat distribution throughout the neck and history of alcoholism. The patient was treated from several diseases associated with alcoholism and hospitalized several times, but the diagnosis of Madelung's disease was omitted. The thyroid gland disease was excluded, while enlargement of the neck adipose tissue was attributed to obesity. *Conclusions*. This study points out possible diagnostic mistakes when a physician is not aware of a differentiation diagnosis of symmetrically enlarged neck masses, especially in geographic regions with high incidence of this disease.

## 1. Background

Multiple symmetric lipomatosis (MSL) also known as Madelung's disease is a rare disorder characterized by the growth of nonencapsulated masses of adipose tissue [1, 2]. The disease was first described by Brodie in 1846 and later by Madelung and Launois in 1888 and 1898, respectively [3]. It is a slow progressing disease with unknown etiology. Madelung's disease is often associated with other disorders such as liver disease, abnormal glucose tolerance, hyperuricemia, hypothyroidism, hypertension, and hyperlipidemia [4, 5]. About 90% of patients have history of alcohol abuse [5, 6]. Madelung's disease has been also related, though very rarely, to the occurrence of malignant transformation of the fat tissue and oropharyngeal tumors [7]. Unlike simple obesity, which is characterized by general increase in total body fat, in Madelung's disease fat masses are present in the face, neck, occipital fossa, and shoulder areas. Fat masses are symmetrically distributed, while distal arms and legs are spared [8]. At first, patients usually complain on aesthetic changes. Later, most patients with Madelung's disease suffer from reduced neck mobility or compression of respiratory structures. Increased fat masses can compress surrounding tissues such as nerves, vessels, and neck muscles thus causing reduction of size of trachea and esophagus leading to the obstruction of the upper respiratory pathways or causing obstructive sleep apnea [9, 10]. Other clinical manifestations include progressive myopathy, laryngeal obstruction, and polyneuropathy. However, the most common and prominent clinical sign is symmetrical distribution of the fat masses in the neck area [11–13].

 Here, we present the case of Madelung's disease with symmetric fat distribution throughout the neck and history of alcoholism. The patient was treated from several diseases associated with alcoholism and hospitalized several times, but the diagnosis of Madelung's disease was omitted. The thyroid gland disease was excluded, while enlargement of the neck adipose tissue was attributed to obesity. This study stresses possible diagnostic mistakes when a physician is not aware of a differentiation diagnosis of symmetrically enlarged neck masses, especially in geographic regions with high incidence of this disease.

## 2. Case Presentation 

A sixty-year-old man was admitted to a hospital because he had fever, dyspnea, cough, and chest pain. The clinical and laboratory exams and chest X-ray confirmed pneumonia. The patient completely recovered after an antibiotic treatment.

**Figure 1:**
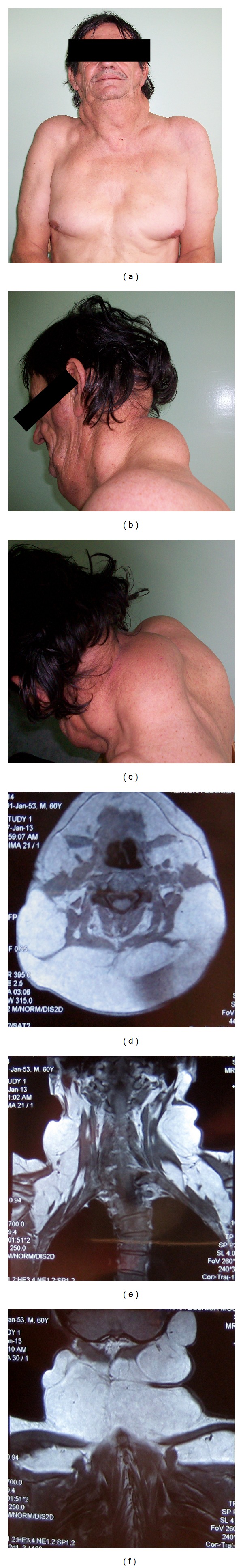
Picture of the patient showing Madelung's disease with symmetric accumulation of lipomatous tissue in the cervical region. The most prominent enlargement was found in the retroauricular, occipital, and upper back areas ((a)–(c)). A cervicothoracic magnetic resonance imaging of the neck showing bilateral subcutaneous masses of nonencapsulated adipose tissue in ventral and lateral regions of the neck ((d)–(f)).

 The patient reported more than 25 years of alcohol abuse as well as smoking two packs of cigarettes a day and recent hospitalization due to acute pancreatitis. His family history was unremarkable for neoplasms and congenital diseases. Before this hospitalization, he was examined because of suspicion on thyroid gland disease. Also, for some time, he has been taking medications for chronic heart failure disease. He stated slow growth of a neck mass in the last 20 years. Physical examination showed a bilateral enlargement of the retroauricular, occipital, and upper back areas. Patient was 178 cm tall and weighted 76 kg his body mass index was normal (24.0 kg/m^2^). Laboratory blood analysis revealed elevated concentrations of aspartate aminotransferase (195 U/L), alanine aminotransferase (290 U/L), gamma glutamyltransferase (440 U/L), and C-reactive protein (24.3 mg/L). The blood triglycerides and cholesterol were within normal range. All other laboratory findings were in reference ranges. Based on clinical presentation and patient's history of long time alcohol abuse, the working diagnosis of Madelung's disease was established. The confirmation was made by magnetic resonance imaging (MRI). A cervicothoracic MRI showed an accumulation of large, bilateral subcutaneous masses of nonencapsulated adipose tissue in ventral and lateral regions of the neck and especially in a suboccipital region, pushing against the atrophic neck muscles typical of Madelung's disease (Figure 1). Excision biopsy of the neck adipose mass confirmed adipose tissue mass without malignant transformation. The patient did not have any respiratory or other symptoms associated with Madelung's disease, and therefore, the surgical treatment was not performed. The patient was advised to cease alcohol consumption and to visit the doctor regularly in order to monitor progression of the disease.

## 3. Discussion

The etiology of Madelung's disease is unknown. Recent studies suggest that mitochondrial disorder of brown fat tissue, or a defect in the adrenergic stimulated lipolysis, is involved in the etiology of this disease. The disease mostly occurs in white males (male : female 15 : 1) of Mediterranean and eastern European population aged from 40 to 50 years [8, 14, 15]. Although the disease incidence is unknown, it is considered that some populations have higher incidence, for example, the incidence rate for males in Italy is estimated to be 1/25 000 [16]. The diagnosis is usually made based on clinical presentation, disease history, and computed tomography (CT) or MRI. Routine chest radiographs may show abnormal symmetrical adipose mass accumulation. MRI is the best diagnostic tool in evaluation of the spread of adipose tissue, presence of tracheal compression, vascular topography within the fat mass, and exclusion of synchronous malignant disease [17]. However, overlap in a clinical appearance with other diseases can sometimes be misleading. For example, the characteristic symmetrical distribution of lipomas may lead to misdiagnosis of Madelung's disease as obesity. Therefore, to confirm definitive diagnosis, biopsy of fatty mass should be performed. The typical distribution consists of massive lipomatous deposits around the neck, which gives the classic descriptions of lipoma as anulare colli, “buffalo hump”, and “horse collar.” Madelung's disease is characterized by diffuse, symmetric, painless, nonencapsulated, and irreversible growth of lipomatosis [14]. One of the possible reasons for misdiagnosis of this disease is probably the fact that there are no strict inclusion criteria as to the localization and dimensions of lipomas in Madelung's disease because reports are scarce in the literature. One of the most significant criteria remains the absence of a capsule together with typical localization [8]. As the disease progress slowly, the delay in diagnosis and misdiagnosis are possible in the first phase of the illness, when the symptoms and signs are not conspicuous, until the occurrence of grotesque appearance. In our case, the patient was treated from pancreatitis, pneumonia, and lesion of the liver, and for some time he has been examined because of suspicion of thyroid gland disease. Later, the adipose tissue enlargement was attributed to the adiposity. However, the typical clinical presentation and patient's history of long-term alcohol abuse were not taken into account. During hospitalization in our institution, diagnosis was made based on clinical presentation and patient's history, while definitive diagnosis was confirmed by MRI showing symmetrical nonencapsulated adipose mass and neck muscle atrophy and by excision biopsy of the neck adipose mass, which showed adipose tissue mass without inclusions of other cell types. The treatment of Madelung's disease is unsatisfactory. Weight loss and cessation of alcohol consumption basically have no effect on growth of the lipomatous masses. Also, there is no effective medical treatment, while liposuction is proposed in the patients with smaller masses. Surgical excision is a treatment of choice in the patients with larger masses or with severe cosmetic deformities and compression syndromes [14]. There is often a recurrence of the masses after liposuction and excision [8].

## 4. Conclusion

Increasing awareness of the presence of this rare disease in our medical community, as a geographic region with high incidence, could prevent misdiagnosis and management misleading. Unique appearance of Madelung's disease should make differential diagnosis simple, especially when physicians are aware of this condition. Moreover, the postponement of diagnosis is most pronounced if a physician is not informed about this pathologic entity and when simultaneous diseases complicate clinical presentation. Therefore, large, symmetrical, and bilateral enlargements in head, neck, and shoulders regions should be considered in sense of Madelung's disease especially in geographic regions with higher incidence such as Mediterranean basin.
